# Long-term CT follow-up of patients with lumbar spondylolysis reveals low rate of spontaneous bone fusion

**DOI:** 10.1007/s00256-024-04650-2

**Published:** 2024-03-21

**Authors:** Anita Staudenmann, Adrian Alexander Marth, Christoph Stern, Stefan Fröhlich, Reto Sutter

**Affiliations:** 1 Department of Radiology, Balgrist University Hospital, University of Zurich, Forchstr. 340, Zurich, Switzerland; 2 Swiss Center for Musculoskeletal Imaging, Balgrist Campus AG, Zurich, Switzerland; 3 Department of Sports Medicine, Balgrist University Hospital, University of Zurich, Forchstr. 340, Zurich, Switzerland

**Keywords:** Lumbar spondylolysis, Bone fusion, Follow-up

## Abstract

**Objectives:**

Knowledge about the long-term outcome of patients with lumbar spondylolysis (LS) is limited. This study assessed the frequency of bone fusion in conservatively treated lumbar spondylolysis with photon counting detector computed tomography.

**Methods:**

Patients with lumbar spondylolysis diagnosed with CT or MRI were prospectively enrolled and underwent CT 5–10 years after initial imaging. Image assessment included evaluation of Meyerding grade, listhesis size, measurement of the lysis gap, and disc integrity on the lysis level. Comparisons were made between bone fusion as the primary endpoint and sex, body mass index, age at diagnosis, follow-up interval, size of listhesis, Meyerding grade, size of the lysis gap, sports activity, and presence of pain.

**Results:**

A total of 39 patients (26.0 ± 3.1 years, 15 female) with lumbar spondylolysis on 41 levels were included after a mean follow-up period of 9.1 ± 2.2 years. Nine patients (22.0%, four female) showed complete fusion of the lysis gap. Patients with bone fusion of the lysis gap had a significantly lower Meyerding grade (*p* = 0.01), smaller size of the listhesis (*p* = 0.019), and smaller anterior and posterior lysis gap size (*p* = 0.046 and *p* = 0.011, respectively). Unilateral lyses showed significantly higher fusion rates than bilateral lyses (40.0% vs. 16.1%, *p* = 0.01). No statistically significant difference was found for pain at follow-up between patients with and without bone fusion (*p* = 0.253).

**Conclusion:**

Bone fusion occurred in about a fifth of conservatively treated lumbar spondylolysis after a follow-up period of 9 years. Factors associated with a successful fusion were a lower Meyerding grade, minimal listhesis, and a small lysis gap.

## Introduction

Lumbar spondylolysis (LS) is a common spinal condition characterized by a defect or stress fracture in the vertebral arch of the spine, more specific in the pars interarticularis [1]. It is most frequently observed in the lower lumbar spine, particularly the fifth lumbar vertebra (81%) with the incidence decreasing significantly in the vertebrae above [2]. The defect is often bilateral; unilateral spondylolysis occurs in only about 16% [3].

The overall prevalence of LS in the general population is estimated to be 6–8%, while the majority of cases of LS are thought to be asymptomatic, making it an often unrecognized condition [4]. A genetic predisposition with a weak pars interarticularis is one of the suspected causes of LS, and it rarely occurs after a single acute trauma [5–7]. LS is particularly common in young athletes, with a higher incidence observed in adolescents participating in activities that involve repetitive hyperextension and rotation of the spine such as gymnastics, football, and weightlifting [2, 8].

LS leads to instability between the anterior and posterior parts of the vertebra. Since the progression of spondylolysis to spondylolisthesis occurs most frequently in adolescents younger than 16 years of age [6], a study by Farfan et al. suggested [9] that the origin might be the mechanically frailest site between two vertebrae during the growth period, which is the growth zone closely associated with the vertebral endplate and intervertebral disc. Based on these observations, a biomechanical pathomechanism for the development of spondylolisthesis was proposed by Sairyo et al. [10]: Initially, spondylolysis alters spinal kinematics, leading to stress accumulation at the growth plate during lumbar movement. Over time, the load accumulation may lead to a physis stress fracture at the vertebral body and ultimately cause spondylolisthesis.

Estimated prevalence of spondylolisthesis caused by bilateral LS is about 3.1% and the segmental instability is also responsible for increased degeneration and disc pathologies at the lysis level [11, 12].

Conservative management is a widely accepted approach for the treatment of LS. This approach focuses on pain relief, activity modification, physical therapy, and bracing, aiming to alleviate symptoms and promote healing of the bone defect [13, 14]. Surgery is rarely necessary for LS and is mostly done in patients with consecutive listhesis, which then leads to foraminal stenosis and radiculopathy [13, 15–17].

While there is substantial knowledge about LS, the long-term results of conservative treatment of spondylolysis, particularly with regard to bone fusion, have remained a subject of debate and interest [18, 19].

Therefore, we set out to conduct a prospective study to shed light on the long-term follow-up of conservatively treated spondylolysis and evaluate the frequency of bone fusion.

## Materials and methods

### Study participants

In this prospective study conducted at a single center, patients of the local institution with clinical diagnosis of LS between 2013 and 2016 and available cross-sectional imaging (MRI/CT) were included in this study. Approval was obtained from the local ethics committee. All examinations were performed in accordance with the Declaration of Helsinki. Informed consent was obtained in advance from each participating patient. MRI scans were performed on 1.5 and 3 Tesla systems (Magnetom Avanto^fit^ / Magnetom Skyra^fit^, Siemens Healthineers), while CT scans were performed on an energy integrating detector CT system (Somatom Definition AS, Siemens Healthineers) according to the local standard clinical protocol. The scan protocol for CT and MRI at the time point at diagnosis can be found in Tables 1 and 2. Further inclusion criteria were a conservative treatment and an age of 18 to 30 years at follow-up. The upper age limit was chosen to maximize the likelihood that spondylolysis, rather than incipient degeneration, was the cause of symptoms in patients who were still symptomatic. Exclusion criteria were an age below 18 years, pregnancy, lumbar surgery, intake of bisphosphonate medication, or an underlying hematological disease. Women were tested for pregnancy before the follow-up CT scan.

**Table 1:** Protocol used for tin-prefiltered CT

Parameter	CT at diagnosis	Follow-up CT
Tube voltage (kVp)	100	140
Reference tube current-time products (mAs)	182	134
Collimation width (mm)	144 × 0.4	144 × 0.4
Pitch	0.8	0.8
Rotation time (s)	1.0	1.0
Field of view (mm)	160	160
Focal spot (mm)	0.8 × 1.2	0.8 × 1.2
Matrix size	512 × 512	512 × 512
Slice thickness (mm)	2	2
Increment (mm)	2	2

*kVp* kilovoltage peak, *mAs* milliampere-seconds

**Table 2:** Sequence parameters for the clinical protocol of 1.5 T and 3.0 T MRI scans

	T2 TSE (sagittal)	T1 TSE (sagittal)	T2 TSE (axial)	STIR (sagittal)
Repetition time (ms)	4000	550	3500	4450
Time to echo (ms)	90	12	115	63
Inversion time (ms)	–	–	–	150
Field of view	300 × 300	300 × 300	220 × 220	300 × 300
Matrix size	512 × 512	512 × 512	512 × 512	384 × 384
Slice thickness (mm)	4.0	4.0	4.0	4.0

*STIR* short tau inversion recovery, *TSE* turbo spin echo

### Clinical data

Prior to the follow-up CT scan, each participant completed a questionnaire regarding clinical symptoms and sports activities (Appendix). Numerical Rating Scale (NRS) ranging from 0 to 10 was utilized to evaluate numbers of days with pain per week at the time of diagnosis and follow-up. Further questions included type of conservative therapy, involvement in sports activities, and main type of sport. Additionally, patients were asked if the sport was performed competitively and whether the sport had to be stopped, reduced in intensity or if the activity level could be maintained despite the diagnosis of LS.

### Follow-up CT imaging

For the follow-up CT, all participants underwent non-contrast imaging on a clinical photon counting detector scanner (Naeotom Alpha, Siemens Healthineers) that covered the affected vertebra and its adjacent vertebral segments above and below. Tin prefiltration was used as part of the clinical imaging routine to decrease the radiation dose while maintaining image quality, which has been previously described by other authors [20, 21]. The objective of the study was to assess the natural lumbar progression of lumbar spondylolysis over an extended follow-up period; the choice of the CT scanner and the use of tin filter was done to obtain CT images with as little radiation dose as possible, but the specific CT technique was not part of the study hypothesis. Patients were positioned supine with feet first on the table of the CT scanner. The scan protocol is summarized in Table 1. CT dose index (CTDI_vol_) for the follow-up CT was automatically obtained using a commercial dose management and reporting platform (EasyDoseQM, v1.6.138, BMS Informationstechnologie GmbH).

### Image analysis

The datasets from the initial imaging examination and the follow-up CT were evaluated by two radiologists (A.A.M. and C.S.) with 4 and 8 years of experience in musculoskeletal radiology independently on a dedicated picture archiving and communications system workstation in a randomized fashion and blinded to all clinical information. If their opinions differed, the final description was determined by a third observer (R.S.) with 18 years of experience in musculoskeletal radiology. From 2013 to 2016, MRI served as the institutional standard for diagnosing LS. In cases in which multiple MRIs of a patient were available, the one in which the lysis was initially diagnosed was evaluated. In six cases, the initial diagnosis was based solely on CT scans and no MRI scans were performed.

**Fig. 1:**
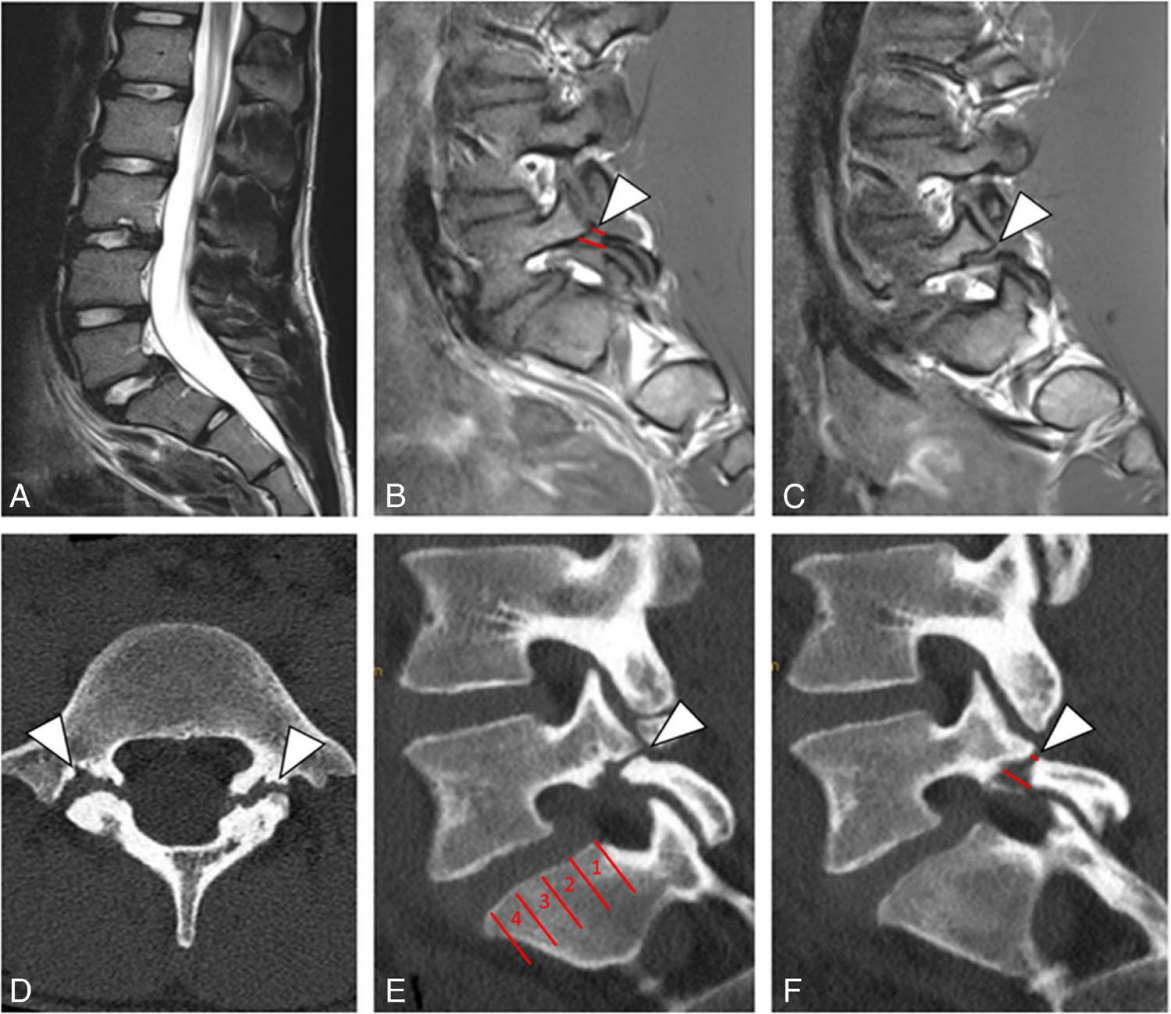
**A** Baseline sagittal T2-weighted MRI image of a 17-year-old male shows anterolisthesis Meyerding grade I at the level L5/S1 with an anterolisthesis distance of 6 mm. **B** T1-weighted sagittal MR image on the left side with lysis gap (arrowhead). Red lines indicate the measurement of anterior and posterior lysis gap at the level of the facet joints. **C** T1-weighted sagittal MR image on the right side with lysis gap (arrowhead). **D** Axial follow-up CT image of the same patient (now 24 years old) with persisting bilateral lysis (arrowheads). **E** Sagittal follow-up CT image on the left side with persisting lysis gap (arrowhead). The severity of spondylolisthesis was graded according to the Meyerding classification, dividing the slippage in relation to the quarter of the superior endplate of the vertebra below, and **F **sagittal follow-up CT image on the right side with lysis gap (arrowhead). Red lines indicate the measurement of anterior and posterior lysis gap

**Fig. 2:**
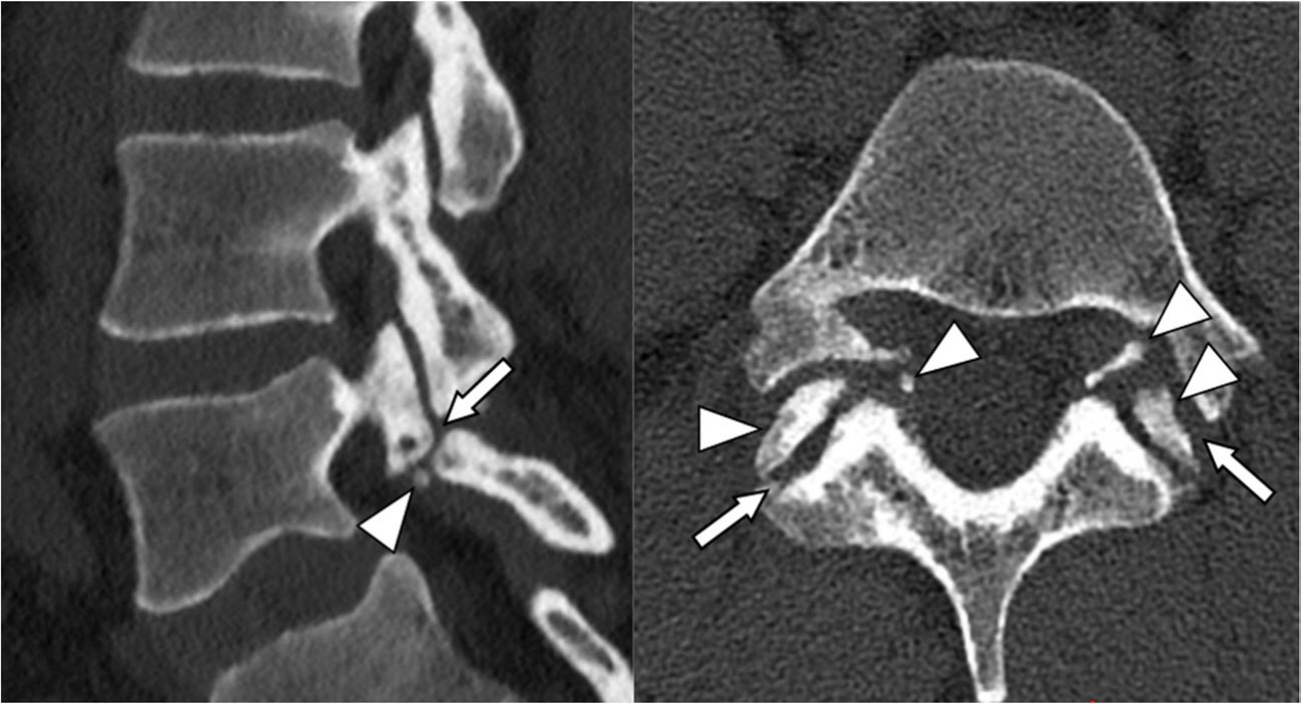
Sagittal left follow-up CT image of the lumbar spine and axial image of a persisting lysis gap (arrow) on the left and right side and multiple ossicles in the lysis gap (arrowheads) in a 23-year-old female patient

The following parameters were assessed on the initial imaging examination at the time of diagnosis, as well as on the prospectively acquired follow-up CT:

1. Spondylolysis and lysis gap: Axial and sagittal images were analyzed to report presence or absence of spondylolysis bi- or unilaterally. If present, the lysis gap was measured in mm at the anterior edge and posterior edge on sagittal images at the level of the facet joints (Fig. 1). Also, the presence of one or multiple ossicles in the lysis gap of the spondylolysis was reported (Fig. 2). Osseous fusion of the spondylolysis was defined as a continuous pars interarticularis with a well-developed trabecular structure.
2. Spondylolisthesis and Meyerding grading: Anterolisthesis was measured in mm in the sagittal plane and graded according to the Meyerding classification [16].
3. Disc degeneration: Disc protrusion and disc bulging were evaluated in the axial plane. Protrusion was defined as the base of the herniated disc being wider than the dome, whereas bulging was defined as an outward displacement of disc material involving more than 25% of its circumference [22].
4. Bone stress reaction: This was defined as presence of bone marrow edema adjacent to the lysis gap.

### Statistical analysis

SPSS Statistics software (v25, IBM Corp.) was utilized for all statistical analyses. Descriptive statistics were employed to analyze and summarize the characteristics of the study group. The normal distribution and homogeneity of variances for continuous variables were assessed using the Shapiro-Wilk test and Levene’s test, respectively. To evaluate group differences in continuous variables, ANOVA or the Kruskal-Wallis test was employed. For categorical variables, group differences were determined through chi-squared analysis. In case of significant results, post hoc pairwise comparisons were conducted using the Bonferroni correction method. Statistical significance was considered at an alpha level of less than 0.05.

The inter-reader agreement for continuous data among different readers was assessed using intraclass correlation coefficients (ICC) [23] and Cohen’s Kappa for categorical data [24]. Level of agreement was reported according to Landis et al. [24] (≥ 0.8: almost perfect; 0.61–0.8: substantial; 0.41–0.6: moderate; 0.21–0.4: fair; ≤ 0.2: poor).

## Results

### Patient characteristics and radiation dose

**Fig. 3:**
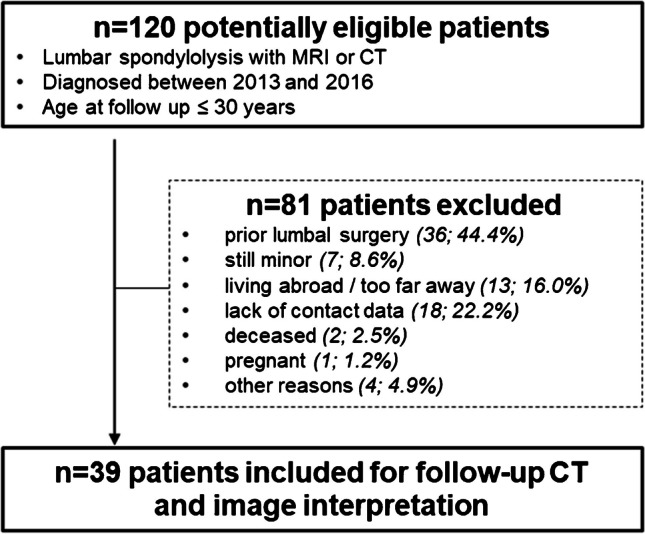
Patient selection flow chart

The database search revealed 120 possible patients who received the radiological diagnosis of LS by means of cross-sectional imaging (MRI/CT), of which 39 agreed to participate in the study and underwent CT at follow-up (Fig. 3): 24 (61.5%) male and 15 (38.5%) female. By the time of diagnosis, 69.2% (*n* = 27) were 18 years old or younger, average age was 17.0 years (± 3.1). The average age at time of follow-up CT was 26.0 years (± 3.1). Follow-up CT was performed on average 9.1 years (± 2.2) after diagnosis. Average BMI of the study population was 23.0 (± 4.6). Since one participant had multiple lyses on three segments (L3–L5), in total 41 lyses were evaluated. All demographic data are summarized in Table 3. CTDI_vol_ was 4.37 ± 1.01 mGy at follow-up CT imaging and 8.16 ± 1.85 mGy in the six cases in which only CT was available for the initial LS diagnosis.

**Table 3:** Demographics of all 39 patients included in the study

Variable	Mean (SD)
Male (*n*, %)	24 (61.5)
Female (*n*, %)	15 (38.5)
Age at time of diagnosis (years)	17.0 (± 3.1)
Age at time of follow-up CT (years)	26.0 (± 3.1)
Follow-up interval (years)	9.1 (± 2.2)
Weight (kg)	67.9 (± 18.6)
Height (cm)	170 (± 11.4)
BMI (kg/m^2^)	23.0 (± 4.6)
Sports at diagnosis (n, %)	33 (84.6)
Thereof competitive	27 (81.8)

*BMI* body mass index, *SD* standard deviation

### LS location and lysis gap

Imaging at the time of diagnosis showed LS in the pars interarticularis of the 5th lumbar vertebra in 78% (*n* = 32), at the level L4 in 17% (*n* = 7), and only in 5% (*n* = 2) at the level L3. Then, 31 LS (75.6%) showed a bilateral defect and 10 (24.4%) a unilateral lysis. Inter-reader agreement for detection of LS was perfect with κ = 1.00 (95% Confidence interval [CI] 0.654–1.00).

Lysis gap on initial imaging showed an average lysis gap at the anterior edge of 2.64 mm (± 1.97; ICC = 0.72 (0.61–0.81)) and at the posterior edge of 2.23 mm (± 2.13; ICC = 0.71 (0.64–0.80)). The values of the same parameters at follow-up CT were anterior 2.23 mm (± 2.87; ICC = 0.89 (0.79–0.96)) and posterior 1.89 mm (± 2.52; ICC = 0.86 (0.72–0.97)). In the group that showed complete fusion in the follow-up, the lysis gap at time of diagnosis was significantly narrower anteriorly (1.37 mm ± 1.06, *p* = 0.046) and posteriorly (0.88 mm ± 0.90, *p* = 0.011) than the average lysis gap in the group that showed persistent lysis (anterior 2.99 mm ± 2.06; posterior 2.61 mm ± 2.09). Persistent bilateral lysis showed larger lysis gaps (anterior 3.22 mm ± 3.37; posterior 3.05 mm ± 2.07) than persistent unilateral lysis (anterior 0.76 mm ± 0.38; posterior 0.68 mm ± 0.88).

### Bone fusion at follow-up

**Fig. 4:**
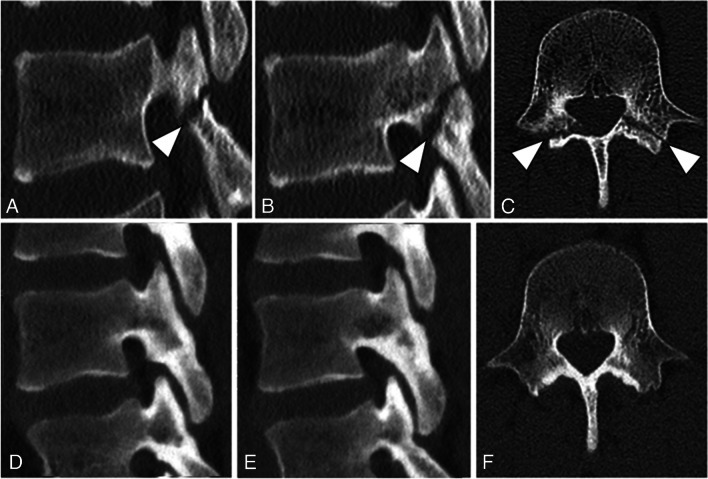
**A** Sagittal base-line CT image on the left side of a 15-year-old adolescent with a spondylolysis of L4 (arrowhead). **B** Sagittal CT image on the right side with a spondylolysis at the same level (arrowhead). **C** Axial CT image of the same patient with the bilateral spondylolysis (arrowheads). **D** Sagittal follow-up CT image of the same patient (now 23 years old) with fused lysis gap on the left side, and **E** sagittal follow-up CT image on the right side with fused lysis gap. **F** Axial follow-up CT image of L4 in the same patient with bilateral fused spondylolysis

A total of nine lyses (five bilateral, four unilateral) showed complete fusion at follow-up (see Table 4 and Fig. 4). Four out of 10 unilateral lyses (40.0%) showed complete fusion, whereas only five out of 31 bilateral lyses fused (16.1%), which was statistically different for both uni- and bilateral lysis compared to bone fusion (*p* = 0.01, Table 5). The likelihood of fusion was investigated in relation to various factors. Statistically significant associations were only found between fusion and Meyerding grade (*p* = 0.01), listhesis size (*p* = 0.019), and the lysis gap measurement anteriorly (*p* = 0.046) as well as posteriorly (*p* = 0.011).

**Table 4:** Fusion rate on different lumbar levels and on bilateral and unilateral lysis

Variable	Initial imaging *n* (%)	Follow-up *n* (%)	Fusion *n* (%)
Lysis level			
L3	2 (5.0)	1 (50.0)	1 (50.0)
L4	7 (17.0)	5 (71.4)	2 (28.6)
L5	32 (78.0)	25 (78.1)	7 (21.9)
Bilateral lysis	31 (75.6)	26 (83.9)	5 (16.1)
Unilateral lysis	10 (24.4)	6 (60.0)	4 (40.0)

**Table 5:** Characteristics of the study population and of different subgroups. Data are given as mean and standard deviation

	Total study population (*n* = 41)	Persisting bilateral lysis (*n* = 25)	Persisting unilateral lysis (*n* = 6)	Complete fusion of lysis (*n* = 9)	*p* value^b^
Sex (% female)	38.5	36	16.7	44.5	0.535
BMI	23.0 (± 4.6)	23.3 (± 4.9)	24.8 (± 6.3)	21.7 (± 3.2)	0.439
Age^a^	17 (± 3.1)	17.2 (± 3.1)	17.8 (± 2.5)	15.4 (± 4.5)	0.273
Follow-up interval (years)	9 (± 2.2)	8.9	8.4	9.11	0.826
Sports activities^a^ (%)	84.6	92	83.3	66.6	0.188
Competitive sports^a^ (%)	81.8	72	66.6	77.8	0.891
Spondylolisthesis, Meyerding ≥°1 (%)	51.2	68	16.6	11.1	0.004*
Listhesis (mm)	2.28 (± 3.05)	3.22 (± 3.37)	0.67 (± 1.63)	0.5 (± 1.5)	0.019*
Lysis gap anterior (mm)	2.23 (± 2.87)	3.51 (± 1.94)	0.76 (± 0.38)	1.37 (± 1.06)	0.046*
Lysis gap posterior (mm)	1.89 (± 2.52)	3.05 (± 2.07)	0.68 (± 0.36)	0.88 (± 0.90)	0.011*
Bone stress reaction adjacent to LS visible on MRI^a^ (%)	29.3	28.0	33.3	33.3	0.743

Asterisks denote statistical significance

^a^At time point of diagnosis. *BMI Body Mass Index*

^b^*p* value by ANOVA in cases of normal distribution, Kruskal-Wallis test in cases of non-normal distribution for continuous variables and by chi-square analysis for categorical variables. Post hoc tests for pairwise comparisons were applied in cases of significance

Between all three groups, non-significant variables included sex (*p* = 0.58), BMI (*p* = 0.439), age at diagnosis (*p* = 0.273), and follow-up interval (*p* = 0.826). Additionally, the study also considered whether the patient participated in sports at the time of diagnosis (*p* = 0.072) and whether this participation was competitive (*p* = 0.599), but no significant association was found for these factors.

### Clinical data

Furthermore, 97.4% (*n* = 38) reported having suffered from back pain at the time of diagnosis. Only in one patient the LS was an incidental finding, and the patient did not have back pain at that time. At diagnosis, patients suffered from back pain an average of 6 days per week (± 1.52 days) with NRS 7/10 (± 1.89). At the time of follow-up, 61.5% (*n* = 24) still reported current back pain. Among the 9 patients who achieved complete fusion, 6 individuals (66.6%) continued to experience ongoing back pain. Within the group exhibiting persistent lysis, 18 out of 29 patients (62.1%) still suffered from back pain at time of follow-up. There was no statistically significant association found between the presence of pain and the fusion process (*p* = 0.253).

On average, back pain at follow-up was present at 1 day per week (± 1.76 days) with a median NRS of 2/10 (± 1.31).

Moreover, 84.6% (*n* = 33) were active in sports at the time of diagnosis. And 81.8% thereof (*n* = 27) practiced competitive sports. Only 14.8% of patients (*n* = 4) were able to continue sports after conservative therapy at the same activity level despite the diagnosis, 29.6% (*n* = 8) had to give up their sport due to the complaints, and 18.5% (*n* = 5) were able to continue the sport, but not at the same level. The remaining 37.1% (*n* = 10) stopped the sport because of other reasons. The most named sports were football, gymnastics, volleyball, and ice hockey.

### Spondylolisthesis and Meyerding grading

At baseline imaging, 21 cases with spondylolistheses Meyerding grade 0 (51.2%), 19 cases with Meyerding grade 1 (46.3%), and one case presenting Meyerding grade 2 (2.5%) were identified, while follow-up CT analysis revealed 20 spondylolistheses with Meyerding grade 0 (48.7%), 20 with Meyerding grade 1 (= 48.7%), and one with Meyerding grade 2 (2.5%). Inter-reader agreement for detection of spondylolisthesis and Meyerding classification was perfect (κ = 1.00 [95% CI 0.715–1.00] and κ = 1.00 [95% CI 0.889–1.00], respectively) [24]. Spondylolisthesis Meyerding ≥ grade 1 was significantly more often found in patients with persistent bilateral lysis (68%) than in patients with complete bone fusion (11.1%, *p* = 0.004) (Table 5).

Ossicles were detected in seven cases during baseline imaging and in 17 cases during follow-up. Patients with LS fusion did not display any ossicles during baseline imaging or follow-up CT scans.

### Disc degeneration and bone stress reaction

Among patients with lysis and a listhesis of ≥ Meyerding grade 1, a significantly higher percentage (77.7%) exhibited disc pathologies compared to those without lysis (26.1%). This difference was statistically significant, with a *p* value of 0.001. Inter-reader agreement for disc degeneration was κ = 0.935 (95% CI 0.845–1.00).

Patients with a larger listhesis demonstrated a higher prevalence of discopathy compared to those with a smaller listhesis. Average listhesis in patients with discopathy was 4.07 mm (± 3.57), while it was significantly smaller in patients without discopathy with only 0.91 mm (± 1.64; *p* < 0.001). Bone stress reaction was visible on initial MRI in a total of 12 cases (6 unilateral, 6 bilateral). No significant difference was found for this finding in patients with and without bone fusion (*p* = 0.143).

## Discussion

Not much is known about the long-term follow-up of patients with conservatively treated LS, and there is some controversy about how often spontaneous bone fusion occurs in patients without surgical treatment. Moreover, most studies have used conventional radiographs at baseline and follow-up when reporting the natural history of LS [5, 25–27] and only one study performed cross-sectional imaging both at baseline and follow-up in 11 patients [27].

Other earlier studies [27, 28] with a long-term follow-up of spondylolysis had their focus exclusively on incomplete lyses, characterized by the absence of complete bone interruption but the presence of abnormalities within the pars interarticularis (negative radiograph but positive bone scan). These investigations have reported healing tendencies for incomplete pars defects ranging up to 67%. We report the largest study to date using cross-sectional imaging to investigate the natural history of LS and performed an analysis of demographic and imaging baseline factors associated with bone fusion at a mean follow-up of 9 years.

Most prior research categorized LS into early, progressive, and terminal stages based on characteristics such as hairline fractures, lysis gap, and presence of ossicles [25, 29]. In contrast, our study quantified the lysis gap in millimeters and found that the lysis gap was significantly larger in patients with persistent lysis than in those with bone fusion.

It is noteworthy that unilateral lyses have not been associated with spondylolisthesis, thereby providing more favorable conditions for fusion compared to bilateral cases [5, 25–27]. In our study, unilateral lyses showed significantly higher fusion rates than bilateral lyses. Our findings with a fusion rate of 16% in bilateral and 40% in unilateral lyses align closely with a radiographic study conducted by Fujii et al. [25] in 2004 with a fusion rate of 41.1% in unilateral and 10.3% in bilateral lyses and also with the study by Morita [5] in 1995, where 62% of unilateral cases and 23% of bilateral cases exhibited fusion. Miller et al. [27] conducted a follow-up study in 2004 where CT was available at baseline and follow-up in 11 subjects: Their data showed partial healing in 29% of bilateral lyses, but no case with complete fusion, whereas all four unilateral lyses showed complete fusion at follow-up. However, because the aforementioned study was conducted with younger athletes, differences from our results must be interpreted with caution. Overall, these findings unveil that most cases of bilateral complete LS do not exhibit a predisposition for spontaneous fusion. This underscores the divergence of pars interarticularis fractures of the lumbar spine from typical bone fractures at other locations, which tend to consolidate under normal conditions.

Sex distribution in spondylolysis studies has traditionally favored males over females, typically reported as a 2:1 ratio [7]. Our study included 24 males and 15 females, resulting in a ratio of 1.6:1. The underlying reason for the male predominance has not been scientifically established yet. Typically, such studies include predominantly young athletic patients. It is noteworthy that soccer is the most frequently mentioned sport in every study [2]. The sex imbalance could be influenced by the male predominance in soccer. However, so far no study, including ours, has been able to establish an association between sex and fusion outcomes.

Despite the persistence of many LS cases, the majority of patients in our study reported substantial reductions in symptoms at follow-up when compared to baseline, both regarding the number of painful days and the intensity of pain decreased among surveyed patients.

Interestingly, persistent pain did not correlate with the presence or lack of bone fusion. This means that for patients with LS there is a perspective of possibly becoming pain-free in the long-term even in case of persistent lysis. Only 14% of our study population were able to return to sports at their pre-injury level, in contrast to other studies reporting return-to-sport rates ranging from 75% to 96% [13, 30].

One of the primary objectives of this study was to identify potential factors at baseline imaging influencing the fusion of LS. We discovered a significant association between the Meyerding grade, the size of spondylolisthesis, and the anterior and posterior lysis gap sizes. We have not found any study that has investigated the probability of fusion in relation to Meyerding grade or lysis gap size. However, our data are confirmed by Fujii [25] who also found that vertebrae without spondylolisthesis at baseline fuse more often. Similar to our study, Morita’s [5] and Fujii’s [25] study did not find a significant association between age at diagnosis and the likelihood of fusion.

Although significant associations between certain factors and fusion were found in this study, it is unfortunate that these factors are not within the direct control of the patient, preventing them from actively shaping the healing process. While this may be disappointing for patients seeking definitive answers, it is crucial for healthcare professionals to communicate this uncertainty and manage patient expectations accordingly. Additionally, the new knowledge about these factors might be considered when discussing whether surgery should be performed or not in a patient.

Our study has limitations. While this single-center study reports on the largest number of patients with spondylolysis and cross-sectional imaging both at baseline and at follow-up, the sample size of 41 spondylolyses is still limited. In the future, prospective studies with larger sample sizes and longer follow-up periods may allow to shed even more light on conservatively treated spondylolysis. In addition, the comparability of quantitative measures between MRI and follow-up CT may be limited due to differences in spatial resolution. Another limitation of this study is the reliance on questionnaires for pain assessment, as responses may be susceptible to memory bias, potentially affecting the accuracy of the information obtained. Finally, the variability of individual training levels of the included patients was not assessed, which may make it difficult to draw definitive conclusions from questionnaire-based assessments of sports activity alone.

In summary, spontaneous bone fusion occurred in about a fifth of conservatively treated LS after 9 years in a young population. Factors associated with a successful fusion were a low Meyerding grade, minimal listhesis, and a small lysis gap.

## Abbreviations

CI: Confidence interval
CT: Computed tomography
ICC: Intraclass correlation coefficients
LS: Lumbar spondylolysis
MRI: magnetic resonance imaging
NRS: Numerical Rating Scale
